# Dr. Austin Flint

**Published:** 1915-10

**Authors:** 


**Dr. Austin Flint,** Jefferson 1857, the noted alienist of N. Y. City, died suddenly of apoplexy at his home, in his 80th year, September 22. His father, the physiologist, was the founder of this journal and he himself was on the editorial staff for a few years.

