# Correction

**Published:** 1915-11

**Authors:** 


In our last issue, we incorrectly stated that the recently deceased Austin Flint of New York was the son of the physiologist. He was himself the physiologist, his father, the founder of this Journal, although interested in physiologic problems, being essentially an internist. We are indebted to Dr. Frank Van Fleet for calling attention to this error which was due to confusion, for the moment, on account of the change of designation of the decedent from Jr. to Sr.

